# 

**Published:** 1891-02

**Authors:** 


CONTENTS:

| | | PAGE |
|---|---|---|
| ORIGINAL ARTICLES: | | |
| | Koch's Method with Tuberculosis. By James Law, F. R. C. S. | 51 |
| | Committee Report. By D. E. Salmen, | 75 |
| | Emasculator. | 77 |
| | Age of the Horse, Ox, Dog and Other Domestic Animals. By R. S. Huidekoper, M. D., Veterinarian. | 78 |
| | Recent Patents. | 84 |
| EDITORIAL: | | |
| | Change Publication | 92 |
| | Back Numbers Wanted | 92 |
| SOCIETY PROCEEDINGS: | | |
| | Indiana Association of Veterinary Graduates | 93 |
| | Ohio State Veterinary Medical Association | 99 |
| | Long Island Veterinary Association. | 102 |
| | Keystone Medical Veterinary Association | 102 |
| | Ontario Veterinary Association. | 105 |

